# Membrane potential accelerates sugar uptake by stabilizing the outward facing conformation of the Na/glucose symporter vSGLT

**DOI:** 10.1038/s41467-023-43119-z

**Published:** 2023-11-18

**Authors:** Farha Khan, Matthias Elgeti, Samuel Grandfield, Aviv Paz, Fiona B. Naughton, Frank V. Marcoline, Thorsten Althoff, Natalia Ermolova, Ernest M. Wright, Wayne L. Hubbell, Michael Grabe, Jeff Abramson

**Affiliations:** 1 Department of Physiology, David Geffen School of Medicine, University of California, Los Angeles, Los Angeles, CA 90095 USA; 2 Stein Eye Institute, University of California, Los Angeles, Los Angeles, CA 90095 USA; 3 Institute for Drug Discovery, Leipzig University Medical School, Leipzig, Germany; 4 Department of Pharmaceutical Chemistry, Cardiovascular Research Institute, University of California, San Francisco, San Francisco, CA 94158 USA; 5 Department of Chemistry and Biochemistry, University of California, Los Angeles, Los Angeles, CA 90095 USA; 6 Present Address: Department of Structural Biology, Van Andel Institute, Grand Rapids, MI 49503 USA; 7 Present Address: Renaissance School of Medicine at Stony Brook University, Stony Brook, NY 11794 USA; 8 Present Address: Hauptman-Woodward Medical Research Institute, 700 Ellicott Street, Buffalo, NY 14203 USA

**Keywords:** Permeation and transport, Structural biology

## Abstract

Sodium-dependent glucose transporters (SGLTs) couple a downhill Na^+^ ion gradient to actively transport sugars. Here, we investigate the impact of the membrane potential on vSGLT structure and function using sugar uptake assays, double electron-electron resonance (DEER), electrostatic calculations, and kinetic modeling. Negative membrane potentials, as present in all cell types, shift the conformational equilibrium of vSGLT towards an outward-facing conformation, leading to increased sugar transport rates. Electrostatic calculations identify gating charge residues responsible for this conformational shift that when mutated reduce galactose transport and eliminate the response of vSGLT to potential. Based on these findings, we propose a comprehensive framework for sugar transport *via* vSGLT, where the cellular membrane potential facilitates resetting of the transporter after cargo release. This framework holds significance not only for SGLTs but also for other transporters and channels.

## Abstract (web summary)

Sodium glucose transporters are critical for maintaining glucose homeostasis. Here, authors provide a detailed framework for how SGLTs utilize cellular membrane potentials to stabilize their substrate accepting conformation leading to accelerated transport.

## Introduction

Sodium-dependent glucose transporters (SGLTs) are crucial for the intestinal absorption and the renal reabsorption of glucose, and thus represent an essential component of a complex physiological system that keeps the plasma glucose concentration within the narrow range of 4–10 mmol/l^1^. Notably, SGLT inhibitors are extensively used to manage life-threatening conditions like diabetes, heart failure, and kidney failure, making them significant players in the multi-billion-dollar pharmaceutical industry^2^. SGLTs are the founding members of the Amino acid Polyamine Organo-cation (APC) family, a large superfamily of membrane transport proteins sharing common structural and mechanistic features (https://www.tcdb.org/). To gain insights into the structure-function relationship of their human counterparts, researchers have extensively studied SGLTs using bacterial homologs from *Vibrio parahaemolyticus* (vSGLT) and *Proteus mirabilis* (SiaT)^3–9^.

The crystal structures of vSGLT reveal a five transmembrane inverted repeat (TMIR) topology, organized into distinct ‘bundle’ and ‘scaffold’ domains (Supplementary Fig. 1). Specifically, the bundle domain comprises TMs 1, 2, 6, and 7 while the scaffold domain consists of TMs 3, 4, 5, 8, 9, and 10. Further subdivisions within the scaffold domain are the ‘hash motif’ (TM 3, 4, 8, and 9) and ‘gating helices’ (TM 5 and 10), which play crucial roles in the transporter’s function and undergo conformational changes throughout the transport cycle. The most widely accepted transport mechanism for TMIR-based transporters in the APC family is the alternating access mechanism wherein the accessibility of the substrate binding site alternates between the cell’s exterior and interior using a ‘rocking bundle’ motion, where the bundle domain pivots against the scaffold domain^10,11^.

Active transport of substrates by Na^+^-dependent transporters is primarily driven by the Na^+^ electrochemical gradient, which is comprised of two essential components: the chemical gradient and the electrical potential. The chemical gradient arises from the difference in Na^+^ ion concentration between the intracellular and extracellular sides of the cell membrane, while the electrical potential arises from the charge imbalance across the cell membrane, giving rise to an electrostatic transmembrane potential (TMP) that provides an electrical driving force which influences transport. This resting TMP varies among different cell types. In humans, TMP ranges from approximately −40 mV to −90 mV, whereas in bacteria, the resting TMP is generally higher and capable of reaching −220 mV during the early exponential phase^12^.

Functional studies have played a pivotal role in understanding the significance of electrochemical gradients in driving transport processes in Na^+^-dependent transporters, as well as other voltage-dependent transporters and channels^1,13,14^. Furthermore, researchers have employed low-resolution structural methods to explore voltage-dependent conformational transitions, which have provided initial insights into how changes in transmembrane potential influence the conformational landscape of these transporters^15,16^. However, detailed high-resolution structural studies focusing on the specific influence of the transmembrane potential on transporters are currently limited, with only a few examples available^17,18^. This limitation arises from inherent challenges in maintaining voltage gradients during experiments conducted using structural techniques such as X-ray crystallography and cryo-electron microscopy.

In this work, we investigate the impact of a negative transmembrane potential on vSGLT using ^14^C-galactose uptake assays, Double Electron-Electron Resonance (DEER), electrostatic calculations, and kinetic modeling. Based on our findings, we conclude that vSGLT utilizes a set of gating charge residues to respond to a negative TMP, stabilizing its outward-facing conformation. Consequently, vSGLT’s substrate binding site becomes more accessible to extracellular sugar, leading to a significant enhancement in sugar transport under cellular TMP conditions. These results provide crucial insights into the influence of a negative membrane potential, which exists in nearly all cell types, on the transport cycle of vSGLT. Understanding these mechanisms is essential for comprehending how TMP can bias and modulate the transport processes in various physiological contexts.

## Results

### Sugar transport is accelerated by negative transmembrane potential

**Fig. 1:**
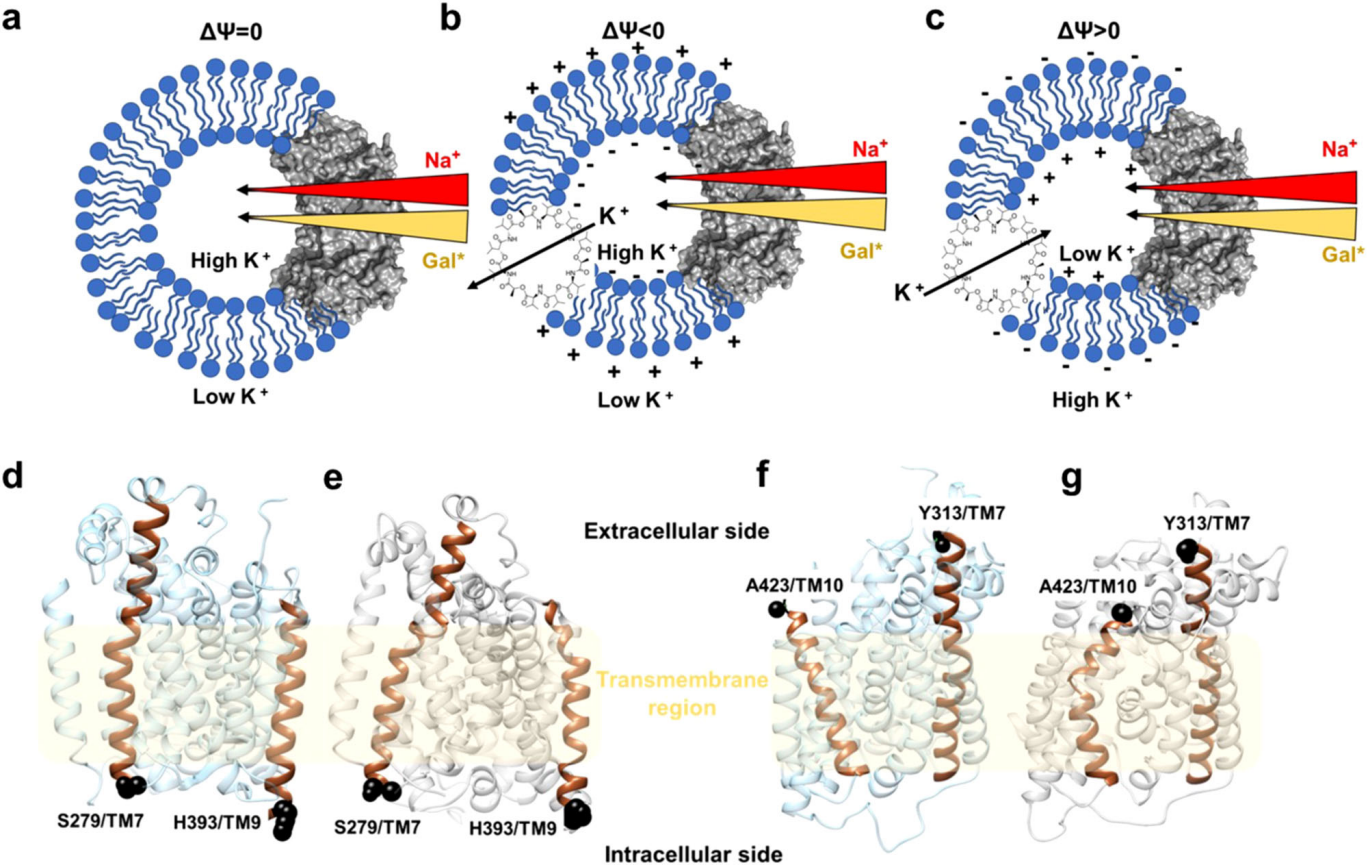
Schematics of voltage generation in a PL system and location of DEER probe sites. In (**a**–**c**), the arrows’ thickness indicates the concentration gradient of Na^+^ (red), ^14^C-Galactose (Gal*, yellow), and K^+^ ions (black). vSGLT is depicted in gray surface representation while lipid molecules are shown in blue. **a** The PL system in the absence of valinomycin is assumed to have no K^+^ diffusional potential. Incorporation of Valinomycin in the PLs leads to K^+^-selective transmembrane diffusion. **b** When [K^+^]_in_ > [K^+^]_out_, a negative potential is established in the PLs. **c** When [K^+^]_out_ > [K^+^]_in_, a positive potential is established in the PL system. **d** The outward open model and (**e**) inward-facing crystal structure of vSGLT (PDB ID: 3DH4) with the chosen sites 279/393 (black spheres) to probe the intracellular side. **f** The outward open model and (**g**) inward-facing crystal structure of vSGLT showing the sites 313/423 on the extracellular side as black spheres.

To investigate the effect of TMP on sugar transport, purified vSGLT was reconstituted into proteoliposomes (PLs). TMP was generated by adding the K^+^-specific ionophore valinomycin to the PLs, which facilitated the selective diffusion of K^+^ ions across the membrane along their electrochemical gradient^19^. We established and modulated membrane potential by varying the concentration of K^+^ ions on the inside or outside of the PLs (Fig. 1a–c and “Methods”), which was confirmed by Oxonol-VI fluorescence (Supplementary Fig. 2)^20^. Additionally, the directionality of vSGLT incorporation into the proteoliposome membrane was assessed, revealing that the incorporation was “mixed” with both orientations (correct and inverted) occurring with equal probabilities (Supplementary Fig. 3 and “Methods”).

**Fig. 2:**
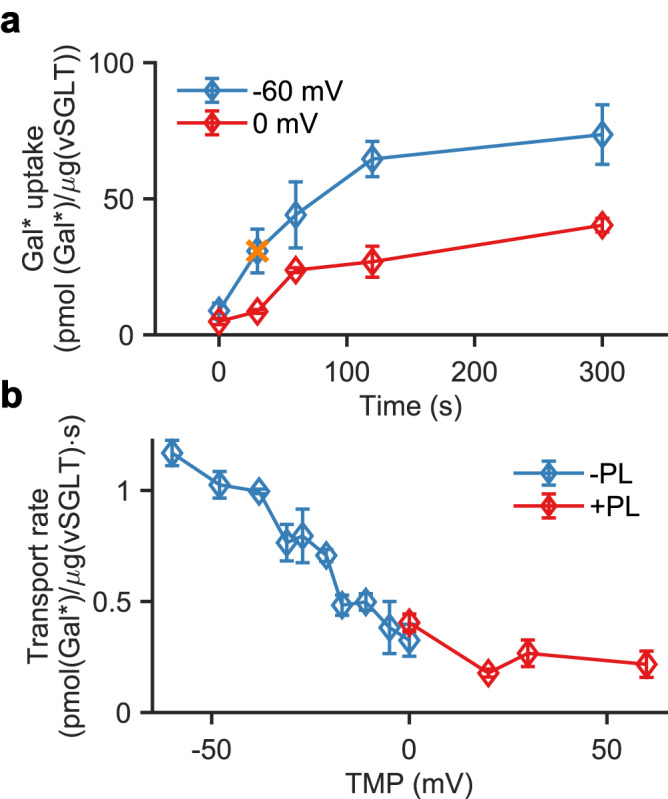
Sugar uptake kinetics. **a** The time course of vSGLT sugar uptake at 0 mV (red), and –60 mV (blue); The 30 s time point is highlighted in orange. **b** The transport rates under the influence of negative (blue) and positive (red) TMPs measured 30 s after TMP generation. The concentrations of Na^+^ and galactose were 73 mM and 28 µM, respectively. Each data point is an average of three independent experiments performed in triplicates. Error bars represent the standard deviation. Source data are provided as a Source Data file.

Next, we examined the influence of TMP on the transport rate of vSGLT using a sugar transport assay with radioactive ^14^C-galactose. The time course of galactose transport exhibited a rapid increase in intraliposomal galactose accumulation, peaking at approximately 5 minutes (Fig. 2a). After the initial period of fast galactose uptake, we observed a subsequent decrease in the net uptake upon dissipation of the Na^+^ gradient (Supplementary Fig. 4a). Importantly, the initial rate of galactose uptake significantly increased from 9 to 35 pmoles/µg/sec when the TMP was decreased from 0 to −60 mV, respectively.

To further characterize the effect of TMP on vSGLT-driven transport, the initial galactose transport rate was determined at various positive and negative TMPs (Fig. 2b). In these experiments, the uptake reaction was terminated after 30 s to ensure transport was unaffected by efflux occurring at later stages of the experiment (cf. Fig. 2a). We found that more negative TMPs resulted in a significant rise in sugar transport rate with a four-fold increase at −60 mV compared to 0 mV. Upon extending the TMP range to positive values, the transport rate showed a significant drop, indicating a non-linear behavior that deviates from the trend observed at negative values (Fig. 2b, red, Supplementary Fig. 4b). While the increased transport rate at negative voltages was expected for a Na^+^-driven transporter like vSGLT, the observed deviation from a linear transport-voltage curve suggests the presence of additional voltage dependence in the protein, similar to what is observed in voltage-gated ion channels^21^.

### Structural basis for membrane potential-induced conformational changes

To explore the impact of transmembrane potential on the structure of vSGLT, we employed double electron-electron resonance (DEER), a pulsed electron paramagnetic resonance technique capable of revealing protein conformational changes *via* distance distributions measured between two spin labels^22^. For this purpose, we generated double cysteine mutants of vSGLT, labeled them with methanethiosulfonate spin-label (MTSL), and incorporated the labeled protein into proteoliposomes to establish and control the transmembrane potential. Conformational changes due to TMP and substrate binding could then be monitored at both the extracellular and intracellular surfaces of vSGLT.

**Fig. 3:**
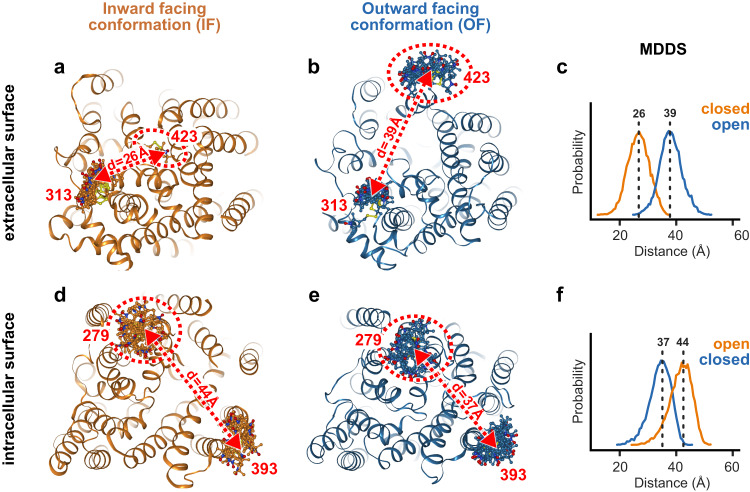
Exploring structural variations between outward- and inward-facing conformations. Tracking extracellular and intracellular conformations of vSGLT via DEER with spin labels at positions 313 and 423 (extracellular side, **a–b**) and 279 and 393 (intracellular side, **d–e**). The red arrows illustrate the distances between spin labels in the inward- and outward-facing conformations. **c** MDDS calculates the distance distributions of the extracellular probes using the inward- and outward-facing conformations depicted in **a** and **b**, respectively. **f** MDDS calculates the distance distributions of the intracellular probe using the inward- and outward-facing conformations depicted in (**d**, **e**), respectively.

To characterize the spin labeling sites used for monitoring the opening and closing of the transporter at the intracellular and extracellular surfaces, we employed the Molecular Modeling of Macromolecules (MMM) software^23^. Specifically, we selected Y313C/A423C on the extracellular surface and S279C/H393C on the intracellular surface to monitor the conformational states of vSGLT. Using Molecular Simulations with Dummy Spin labels (MDDS), we calculated the expected DEER distances for both mutants when residing in the inward-open and outward-open conformations, which were derived from an inward-facing X-ray structure and an outward-facing model^24^, respectively (Figs. 1d-g, 3). The extracellular DEER mutant (313C/423C) exhibited a distance centered around 39 Å in the outward-open conformation and 26 Å in the inward-open conformation, while the intracellular DEER mutant showed a distance of 37 Å in the outward-open conformation and 44 Å in the inward-open conformations (Fig. 3c, f). Thus, the MMM and MDDS calculations confirmed that the chosen spin label pairs exhibited well-separated peaks within the DEER-accessible distance range, enabling us to discriminate the conformational equilibrium on both sides of the transporter.

The ^14^C-galactose uptake assay was employed to assess the functionality of both the extracellular (313C/423C) and intracellular (279C/393C) mutants, confirming their status as functional transporters. Notably, upon labeling with the spin-label, both the extracellular and intracellular DEER mutants showed reduced activity diminishing transport to 14% and 74%, respectively (Supplementary Fig. 5a). Interestingly, the MMM analysis revealed the spin-label side chain at position 423 exhibited a single, constrained conformation when the extracellular side was closed, likely affecting the free energy of this conformation. Accordingly, upon solely spin-labeling position 423C, the transport was reduced to 32% (Supplementary Fig. 5b). However, despite these reductions in overall activity, both constructs showed a significant increase in transport activity in the presence of TMP and were thus suitable monitors to study the effect of TMP on vSGLT by DEER.

**Fig. 4:**
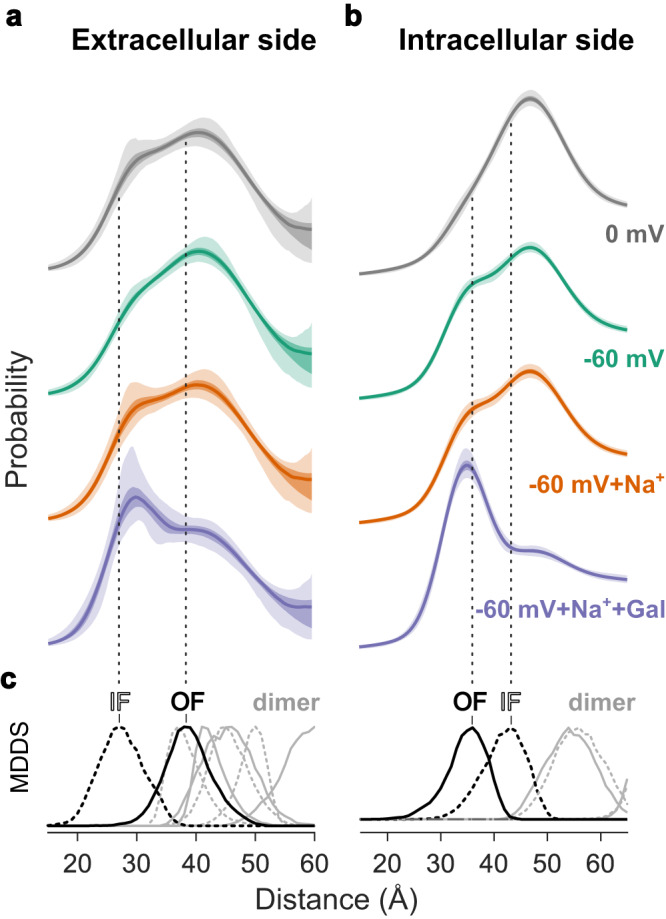
Investigating TMP and substrate-induced structural changes via DEER. **a** DEER distance distributions for the extracellular monitor under different conditions: in the absence of TMP or substrate (gray), in the presence of -60 mV (turquoise), in the presence of –60 mV and Na^+^ (orange), and in the presence of –60 mV, Na^+^, and galactose (purple). **b** Same as (**a**) but for the intracellular monitor. The individual distance distributions have been shifted vertically for clarity. Dipolar evolution data and corresponding fits are available in Supplementary Fig. 8 and the replicate for the extracellular side is presented in Supplementary Fig. 9. The specific distance peaks obtained from MDDS are provided in (**c**), referencing the inward-facing structure (IF, broken line, PDB ID: 3DH4), the outward facing model^6^ (OF) and dimer distances. 50% and 95% confidence bands are shown with increasing transparency. Source data are provided as a Source Data file.

For DEER experiments, we prepared identical spin-labeled vSGLT proteoliposomes and resuspended them in four different buffers to generate the following experimental conditions: (i) 0 mV, (ii) −60 mV, (iii) −60 mV in the presence of Na^+^, (iv) −60 mV in the presence of Na^+^ and galactose. In the absence of TMP (0 mV), our DEER results indicate that both the extracellular and intracellular surfaces exist in an equilibrium between outward- and inward-facing conformations (Fig. 4a–b). Further, the observed DEER distances are in good agreement with the distances obtained from the MDDS calculations (Fig. 4c). In line with previous DEER data on vSGLT solubilized in DDM micelles^7^, the transporter in liposomes shows a preference for the inward-facing conformation in the absence of TMP, where a significant proportion of the population of the extracellular probes reporting a closed conformation and the intracellular probes reporting an open conformation.

In agreement with the constrained spin label side chain at position 423 mentioned earlier (cf. Fig. 3a) and the associated impact on the free energy, the population of the closed extracellular surface is likely underrepresented. Conversely, in the open conformation, the same label explores a significantly greater conformational space, as indicated by the broad peak centered at 39 Å, consistent with the increased dynamics of the extracellular portion of the gating helices (TM9-10) as previously observed in simulations^5, 6^. Additionally, this peak is further broadened by contributions from the vSGLT dimer, as determined by DEER analysis of singly spin-labeled vSGLT (Supplementary Fig. 6 and indicated by the MDDS distances in Fig. 3c).

Under the influence of a −60 mV TMP, the extracellular DEER construct significantly favors the higher distance populations indicative of an outward-open conformation, while the intracellular construct is stabilized in a closed conformation that also agrees well with the outward facing conformation in which the intracellular cavity is closed (Fig. 4, *turquoise*). This demonstrates that TMP causes stabilization of the outward-facing conformation. TMP, when combined with external Na^+^ (*orange*), shows a small population shift towards the closed conformation on the extracellular surface, while the inner gate remains unaffected by the addition of Na^+^. However, it is important to acknowledge that this effect, while intriguing, is not statistically significant at the 95% confidence level and should therefore be interpreted with caution. Finally, the addition of galactose along with Na^+^ under a negative TMP causes the closure of both gates leading to an occluded conformation similar to what has been described for solubilized vSGLT (*purple*)^6,7^. Taken together, our DEER results provide direct evidence for a shift in the conformational equilibrium towards the outward-facing conformation under negative TMP.

### Gating charge calculations identify voltage-sensing residues that drive outward-facing conformation

The concept of “gating charge” is well-known for voltage-dependent changes in ion channels and transporters, where the protein undergoes conformational change(s) to facilitate the passage of ions or substrates. This phenomenon has been well-documented for ion channels and has been observed in studies on SGLT1 expressed in oocytes, using techniques such as electrophysiology and fluorescence^1,13,14^. The uptake data and DEER results obtained in our study provide direct evidence that sugar transport and vSGLT’s conformational state are both influenced by the applied membrane potential. Based on these findings, we postulate that vSGLT responds to transmembrane potential using gating charge residues in a manner similar to voltage-gated ion channels.

**Fig. 5:**
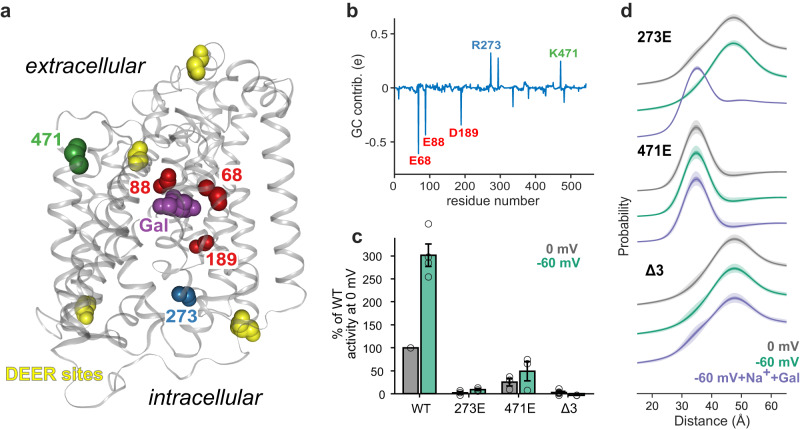
Gating charge calculations and the effect of mutation. **a** Location of the gating charge mutants with respect to substrate (Gal: purple) and DEER labeling sites (yellow). **b** Individual contributions of vSGLT residues to the gating charge calculated from continuum electrostatic calculations using the inward-facing apo structure (PDB ID: 2XQ2) and the outward-facing model. Highlighted residues were further investigated by mutagenesis. **c** Transport activity of GC mutants and their sensitivity to TMP. **d** DEER results of the inner gate gating charge mutants indicate almost complete disruption of the voltage effect in both mutants. However, the R273E mutant still binds substrate leading to closure of the inner gate. The individual distance distributions were shifted vertically for clarity. 50% and 95% confidence bands are shown with increasing transparency. Each data point presented in Fig. 5c is an average of three independent experiments performed as triplicate. Source data are provided as a Source Data file.

To identify putative gating charge residues in vSGLT, we conducted continuum electrostatics calculations on both the inward-facing structure and the outward-facing model which was also utilized for the MDDS calculations (cf. Fig. 3). The continuum electrostatics calculations predicted a modest negative gating charge (GC) value of −0.7 elementary charges (e), which aligns with our observation that negative TMP stabilizes the transporter in an outward-facing conformation. Figure 5a, b showcase the main residues contributing to the gating charge, each providing 0.1 e or more to the charge transfer, and Supplementary Table 4 provides the exact charge contributions for each residue.

### Mutation of gating charge residues abolishes TMP induced conformational changes

Drawing on these calculations, we engineered gating charge mutants of vSGLT by targeting specific residues at different regions of the transporter. These regions include the substrate binding site (Δ3 with E68Q, E88Q, and D189N mutations), the intracellular portion (R273E mutation), and the extracellular portion (K471E mutation). The Δ3 mutant is expected to reduce the absolute value of the GC, thus eliminating vSGLT’s voltage sensitivity and stabilizing the transporter in the inward-facing conformation. On the other hand, the charge reversal of basic residues—R273E and K471E—is predicted to increase sensitivity, facilitating the transition to the outward-facing state under negative voltages.

Assessment of the transport activity of these GC mutants (Fig. 5c) revealed that they all exhibited reduced or no galactose uptake. As anticipated, the charge-neutralizing Δ3 mutant (E68Q, E88Q, and D189N) displayed no activity since these residues are crucial for galactose binding (Fig. 5a/c). The R273E GC mutant also exhibited no activity, possibly due to the guanidinium group’s charge-charge pairings that are hypothesized to be critical for intracellular closure as observed for SiaT. In contrast, the K471E GC mutant demonstrated sugar transport activity, albeit at 20% of the wild-type level. Interestingly, the transport retained voltage sensitivity, showing slightly enhanced uptake at negative TMP.

To further characterize the effect of the GC mutations on the conformational equilibrium, DEER measurements were carried out using the intracellular construct (279C/393C) (Fig. 5d). None of the GC mutants responded to voltage in the absence of sodium and galactose. Initially, we were somewhat surprised by the lack of response from the R273E and K471E mutants, as they were engineered to have more negative gating charge values, which should have made them more voltage sensitive.

For the R273E GC mutant, we observed that it remains in the inward-open conformation at both 0 mV and −60 mV, whereas the wild-type transporter closes the intracellular side under the influence of negative TMP. Upon addition of galactose, both the wild-type and the R273E GC mutant show closure of the intracellular side of vSGLT, indicating that the R273E GC mutant is still capable of binding galactose. As mentioned above, R273 is involved in bridging interactions with other helices, which are destabilized by the charge reversal mutation. It is therefore possible that this mutant still exhibits greater voltage sensitivity; however, the reduced stability of the closed conformation due to the charge reversal impedes gate closure, necessitating a greater negative voltage to surmount the energy barrier for closure. Unfortunately, testing this hypothesis would require extending our spectroscopy measurements to voltages below −60 mV, which is technically not feasible. Nonetheless, galactose binding provides enough energy to overcome this barrier and stabilize the closed conformation.

The equilibrium ensemble of the K471E mutant exhibited a strong shift towards the conformation with the closed inner gate, indicating that this mutant adopts either an occluded or outward-facing conformation even in the absence of voltage and substrate. Inward-facing vSGLT structures, reveal that K471 forms a salt bridge with E429 on the outer-gate helix TM10, and the mutation likely creates charge repulsion, destabilizing the closed outer gate and biasing the transporter towards an outward-open conformation. Since K471E already adopts a closed intracellular conformation at zero voltage, it is difficult to determine if it is more voltage sensitive, as negative voltages would only serve to further stabilize this state of the protein. However, the results from our sugar uptake assays (Fig. 5c) strongly suggest voltage sensitivity and sugar binding capability for the K471E mutant.

As predicted, the Δ3 charge neutralization mutant showed a total loss of voltage sensitivity and substrate binding. It predominantly adopted the inward-facing conformation under all investigated conditions, which explains its inability to carry out transport (Fig. 5c).

These findings highlight the critical role of gating charge residues in finely balancing the equilibrium between inward- and outward-facing conformations of the transporter, both in the presence and absence of TMP. Interestingly, all the GC residues identified in our calculations serve dual functionalities, acting as both GC contributors and substrate binders or GC contributors and gate latches. This observation is not surprising, as gating charge residues are charged, and charged residues often form substrate binding sites and gate closure points, often through salt bridge pairing. Another significant factor is that gating charge residues must move through the membrane’s electric field during the transition. This can occur when they are located on movable parts of the protein (such as the gates) or at regions that influence water accessibility (e.g., along the solute binding pathway) – and all the identified residues fall into one or both of these categories.

## Discussion

### A unifying model integrating transport rates, orientation, and gating charge

**Fig. 6:**
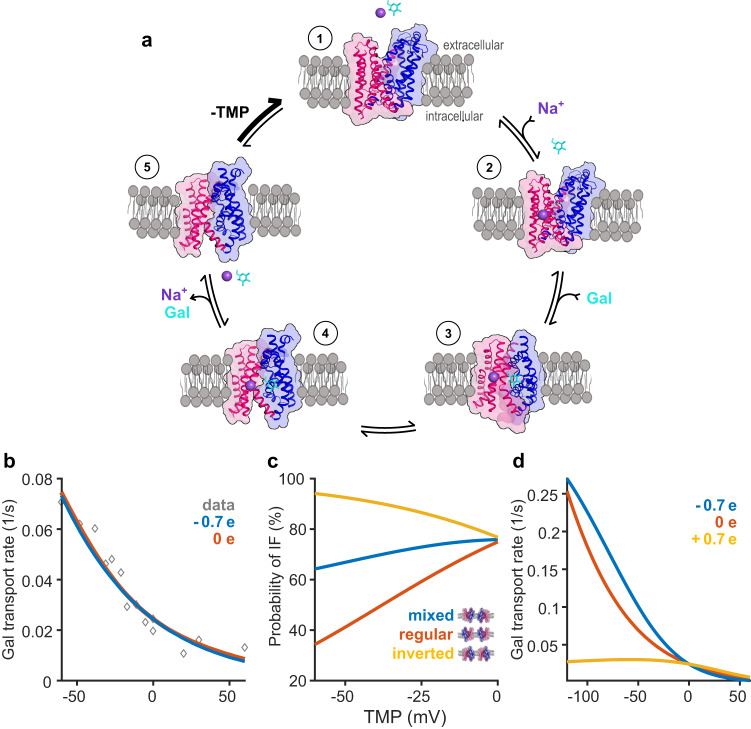
Five-state transport model of vSGLT. **a** A unifying model of a five-state transport cycle for vSGLT based on an aggregate of data^5,7,13,31,50^ now including the effect of transmembrane potential. Outward-facing vSGLT (state 1) binds Na^+^ and undergoes a minor conformational change (state 2). Next, galactose binds and forms an occluded conformation (state 3). vSGLT then transitions to an inward conformation (state 4) where Na^+^ and galactose are stochastically released on the intracellular side (state 5). The inherent transmembrane potential (TMP) drives the conformation of vSGLT back to the outward-facing state where it is primed for another transport cycle. **b** Average transporter turnover rate as a function of voltage in a mixed-population of vSGLT with (Qgc = −0.7e, blue) and without (Qgc=0e, red) gating charge. **c** Steady state probability of inward-facing conformation (sum of states 4 and 5) as a function of voltage for mixed-population of transporters (blue curve), transporters in the inverted conformation (yellow), and transporters in the correct orientation (red) with a gating-charge of Qgc = −0.7e. **d** Single transporter rate of correctly oriented vSGLT with gating-charges of Qgc = −0.7e (blue), 0e (red), and +0.7e (yellow). All calculations in panels b-d use parameters from Supplementary Tables 4, 5 and Na^+^ and galactose concentrations employed from uptake assays.

To integrate our findings from the three complementary approaches, we developed a mathematical model of the transport cycle. Taking into account the results from the gating charge calculations we then used this model to simulate the transport rates and population shifts observed in our DEER experiments. However, the interpretation of both transport and DEER results was complicated by the presence of the two orientations of vSGLT in liposomes: the physiological orientation with the extracellular side facing the external solution, and an inverse orientation with the extracellular side facing the lumen of the proteoliposomes (Supplementary Fig. 3). To address this, we extended our previous mathematical model of the vSGLT transport cycle^5,7^ to accommodate this mixed population of vSGLT in proteoliposomes. The individual steps involved in the standard transport cycle are illustrated in Fig. 6a (for a comprehensive description of the model, please refer to the supplementary information).

By utilizing the Na^+^ and galactose concentrations obtained from the transport assay, we employed kinetic equations across a range of voltages to derive the transporter rates and corresponding steady-state populations along the transport cycle (Fig. 6b). The normalized vSGLT transport rate for the mixed population was calculated based on two assumed protein gating charge values: –0.7 e (blue) and 0 e (red). Both gating charge values yielded similar curves, demonstrating increased transport at more negative membrane potentials and aligning with the transport assay results (data from Fig. 2b shown for comparison). However, the decrease in the inward-facing population at negative potential, observed by DEER, can only be explained by a transporter carrying a negative gating charge (Fig. 6c). At −60 mV, DEER data on the intracellular side revealed an 18 ( ± 4)% reduction in the inward-facing population (from 90% to 72% after accounting for dimer contributions), and the mixed population model predicted a 13% decrease (blue), showcasing a reasonable agreement between the model and experimental data.

Our calculations also predicted changes in the population of transporters incorporated with correct versus inverted orientation. Correctly oriented transporters reached 30% inward-facing (70% outward-facing) at –60 mV, leading to a 40% increase in the outward-facing state relative to 0 mV (red). In contrast, inverted vSGLT molecules exhibited a higher inward-facing population at negative voltages (95% at –60 mV, yellow), consequently reducing the net outward open mixed population as seen in the DEER data (blue). In summary, our comprehensive analysis, combining transport assays, DEER experiments, and kinetic modeling, provides compelling evidence that a negative gating charge is a key factor driving the outward-facing conformation of vSGLT in response to transmembrane potential.

Finally, we used the model to predict the single transporter rate of a correctly oriented protein over a large range of TMPs reaching more negative values characteristic of bacteria (Fig. 6d). In this case, the –0.7 e gating charge enhances the transport rate (blue curve) compared to a transporter with no gating charge (red curve), and even a small positive gating charge value suppresses the transport by a factor of 5–10 for voltages below -100 mV (yellow curve).

Based on this study, we interpret the standard five-state model for the vSGLT transport cycle (Fig. 6a) as follows. In the absence of substrates, the transporter adopts an outward-open resting conformation (state 1), stabilized by the negative TMP and prepared to bind extracellular Na^+^ and sugars. Upon sodium binding to the Na2 site, the transporter transitions to state 2, moderately increasing the propensity for closure of the extracellular vestibule without impacting the intracellular side. Short simulations of our outward-facing model support these localized outer-gate rearrangements, with TM1a and gating charge residue E68 undergoing subtle changes in response to Na^+^ binding (Supplementary Fig. 7). We posit that Na^+^ binding to the Na2 site in vSGLT, as well as hSGLT2, primarily primes the ligand binding site for subsequent sugar binding. Once sugar binds, both gates close, leading to a fully occluded conformation (state 3). The electrochemical potential of Na^+^ influences the isomerization of the transporter to the inward-facing state (state 4), where stochastic release of both Na^+^ and sugar results in the inward-facing apo configuration (state 5). Finally, the membrane potential facilitates the reset of the apo transporter to the outward-facing conformation (state 1), initiating the cycle anew. This comprehensive model provides valuable insights into the intricate transport mechanism of vSGLT, emphasizing the interplay between transmembrane potential, substrate binding, and conformational changes essential for its function.

### Mechanistic implications and concluding remarks

While atomic structures of vSGLT have provided insights into its conformations in the presence and absence of Na^+^ or sugar, the complete dynamic details of the transport cycle remained elusive^4,9,25–27^. Notably, vSGLT exhibits a strong preference for an inward-facing or occluded conformation, as already suggested by our previous DEER studies in detergent micelles^7^. This raises an intriguing question – if vSGLT prefers an inward-facing conformation, how does it transition to an outward-facing conformation during the transport cycle in order to bind its cargo?

Sodium-dependent transporters utilize Na^+^ gradients to facilitate the transportation of substrates, yet the role of transmembrane potential (TMP) has often been overlooked in this context^28^. Voltage-gated ion channels, an extensively studied class of proteins, undergo transitions between open and closed states in response to slight changes in membrane potential. These transitions are governed by a voltage sensor within the protein, characterized by its gating charge^29,30^. Some voltage-gated ion channels exhibit large gating charges (−12 e), granting them exquisite sensitivity, while others, such as the ClC-0 Cl^-^ channel, possess intrinsic gating charges as low as -0.3 e and can still modulate their conformational landscape in response to voltage^31^. However, unlike voltage-gated ion channels, transporters must undergo continuous cycles through a series of conformational states, and strongly favoring one set of states over another may disrupt the entire transport process. We emphasize that the transporter’s gating charge plays a crucial role in the transport cycle by redistributing the electrical component of the electrochemical driving force during the outward-to-inward transition (state 2 → state 4) and the resetting stage (state 5 → state 1). This notion may explain the non-existent or greatly reduced rates imparted by the GC mutants R273E and K471E, respectively, and how these mutants differentially affect the conformational equilibrium in the absence of TMP. Taken together, we propose that transporters have evolved specific gating charge distributions tailored to the TMP and ion/substrate concentration properties of their host cell, enabling them to finely tune transport rates and respond to voltage in more subtle ways than previously understood.

In conclusion, our comprehensive analysis, which combines transport assays, DEER experiments, electrostatic calculations, and mathematical modeling, yields valuable insights into the crucial role of transmembrane potential in vSGLT-mediated sugar transport. We propose a refined five-state model for the vSGLT transport cycle, emphasizing the significance of its -0.7 e gating charge in directing the inward-to-outward transition and stabilizing the outward-open conformation in preparation for a new cycle. The delicate balance of gating charges is indispensable for maintaining efficient transport. While our study primarily focuses on vSGLT and its transport of a single Na^+^ and galactose, we also acknowledge the potential broader impact of these findings on the biophysics of transporters in general.

## Methods

### Protein expression, purification, and labeling for DEER

Expression and purification of wild-type, and mutant vSGLTs were carried out as previously described with a minor change in the buffer used for size exclusion chromatography (50 mM HEPES, pH 7.0, 150 mM NaCl, 0.0174% DDM)^7^. Purified vSGLT was mixed with 20 times molar excess of methanethiosulfonate spin label (MTSL) and incubated in dark with constant stirring for a period of 12 h. After 12 h of incubation, an additional 10 times molar excess of MTSL was added to vSGLT followed by an additional 5 h of incubation. Post labeling, vSGLT was washed in a 100 kDa centricon with 30 volumes of labeling buffer to completely remove the free spin labels.

### Proteoliposome preparation for sugar transport assays and DEER

Dried *E. coli* polar lipids (Avanti Polar Lipids Inc.) were resuspended in water to a final lipid concentration of 20 mg/ml and water-bath sonicated for four minutes. This lipid suspension was mixed with 1% DM and vSGLT at a protein to lipid ratio of 1:120 while maintaining the pH at 7.5 using appropriate buffer as described later in this section. This lipid/protein suspension was incubated for twenty minutes followed by rapid dilution into ice-cold buffer. For inducing negative voltages, the buffer used was 150 mM Potassium Phosphate (KPi), pH 7.5 and for inducing positive voltages, the buffer used was 139 mM Choline Chloride (ChCl), 10 mM HEPES/Tris.HCl pH 7.5, and 1 mM KPi pH 7.5. To harvest the PLs, the solution was spun down at 200,000 × *g*, for two hours, at 4 °C. The buffer was carefully decanted, the tube was thoroughly dried, and the proteoliposomes pellet was resuspended in 150 mM KPi (or other buffer solution, if required) equal to the volume of lipids used in the preceding step. PLs were flash frozen under nitrogen gas and stored at –80 °C until needed. Prior to use, PLs were freeze/thawed six times in a water bath set to 32 °C.

The PLs for DEER measurements were prepared in the same manner with a few additional steps following the freeze/thaw cycle. A protein to lipid ratio (1:100) was used to prepare the PLs to increase the protein content of the PLs and maximize signal for DEER. Following the freeze thaw of the prepared liposomes suspension, the PLs were brought to room temperature and valinomycin was added to the liposomes at a concentration of 4 µM. Next, the PLs were divided into four batches of equal volume and spun at 228,000 g for one hour. The buffer was discarded from the top, and the PL pellet was resuspended in 60 µL of the four DEER buffer conditions supplemented with 4 µM valinomycin. Buffer 1: 150 mM KPi (generating 0 mV), Buffer 2: 150 mM ChCl, 20 mM Tris, pH 7.5 (generating −60 mV), Buffer 3: 150 mM Sodium Phosphate (NPi) (generating –60 mV with Na^+^), and Buffer 4: 150 mM NPi, 20 mM Galactose (generating -60 mV with galactose). These PLs were then loaded into 2.5 mm I.D. borosilicate capillaries. After 4 min the capillaries were flash frozen in a dry ice/EtOH mix and then transferred to a liquid nitrogen dewar until the DEER experiment.

### Sugar transport assay

Sugar transport reactions were initiated by mixing 4 µL of PLs with the appropriate volume of uptake buffers to vary the external K^+^ ion concentration and generate desired voltages (see Supplementary Tables 1 and 2). Reactions were stopped at the indicated time by pipetting 1 mL of ice-cold 150 mM KPi pH 7.5 onto the mixture, rapidly filtering, and washing with 4 mL of the same buffer. Filters were dissolved in 900 µL ethyl acetate, then mixed with 3 mL Ultima Gold Scintillation Cocktail and vortexed. Samples were measured using a Beckman LS 6000IC liquid scintillation counter. As a control, the uptakes were carried out in buffers with NPi replaced by KPi to demonstrate that the PLs are sealed throughout the experimental timeframe and the transport we observe is solely driven by a Na^+^ electrochemical potential gradient. These control values were subtracted from their test set before plotting the traces. All the data are plotted as the mean and standard deviations calculated from three trials of independent triplicates.

### Determining the orientation of vSGLT in PLs

The orientation of vSGLT incorporated into PLs was ascertained by introducing a single reactive cysteine either on the extracellular side (S313, TM7) or on the intracellular side (H393, TM9) of the transporter. vSGLT S313C and H393C in 20 mM Tris pH 7.5, 0.0174% DDM, and 25 mM NaCl were treated with pyrene maleimide at a 10-fold molar excess for 60 min at 4 °C with gentle shaking in the dark. The unreacted label was separated from the protein by a PD-10 desalting column and the labeled protein was reconstituted into PLs as previously described. For quenching experiments, labeled PLs were mixed with different equiosmolar concentrations of KI and KCl (see Supplementary Table 3 for the different compositions) in the presence of the reducing agent Na_2_S_2_O_3_ that prevents the formation of the reactive membrane impermeable I_2_ species. Fluorescence emission levels between 360 and 500 nm were measured using a Fluorolog 3 fluorometer using an excitation wavelength of 340 nm and 4 nm slits. Quenching was quantified using the Stern-Volmer relationship (Eq. 1):

1 $$\frac{{F}_{0}}{F}=1+{K}_{D}[Q]$$

where F_0_ and F are the fluorescence intensities at 378 nm in the absence and presence of a quencher, respectively. K_D_ is the Stern-Volmer quenching constant, and Q is the concentration of quencher.

### Validation of TMP using Oxonol VI fluorescence

Fluorescence measurements were taken on a Fluorolog-3 spectrofluorometer using an excitation wavelength of 580 nm with a 10 nm slit opening, and the emission was recorded between 600-700 nm with a 5 nm slit opening. For the various measurements, 40 nM Oxonol VI in a buffer with appropriate potassium concentration was used. The experiment was carried out by adding 5 µl vSGLT PLs and valinomycin at a final concentration of 20 µM into the oxonol containing buffer and recording the emission spectra as described above. Fluorescence emission values at 630 nm for the samples with valinomycin (F2) were divided by the emission values at 630 nm of the PL samples (F1) devoid of the ionophore. All ratios are plotted as the mean and standard deviations calculated from three different runs of independent triplicates. The voltages shown were calculated using the Nernst potential equation (Eq. (2)):

2 $$E=\frac{{RT}}{{zF}}{{{{\mathrm{ln}}}}}\,\frac{[{{K}^{+}}_{{out}}]}{[{{K}^{+}}_{{in}}]}\,$$

where E is the electric potential, R is the universal gas constant, T is the temperature in Kelvin, z is the ion charge, and F is the Faraday constant.

### DEER measurements and analysis

Four-pulse Q-band double electron-electron resonance was performed at 50 K on a Bruker e580 equipped with an arbitrary waveform generator, 150 W TWT amplifier, and a QT-2 resonator (Bruker, Rheinstetten – Germany). Pulse lengths of π/2 and π pulses were optimized via nutation experiments and set to 16 ns and 32 ns, respectively. A chirp pulse (50 MHz sweep width) was applied 70 MHz above the observer frequency, which itself was set between the two maxima of the nitroxide absorption spectrum. We used 16-step phase cycling and a repetition rate of 510 µs.

DEER data analysis was performed using the DEERLab toolbox^32^ implemented in Matlab (v2019b). Datasets of double mutants were first subjected to power scaling^33^ and then analyzed globally (for 313C/423C including repeats, for 279C/393C including all GC mutants) using multi-Gaussian parametric fitting models including mean distances and FWHMs as global parameters, and fractions (areas of the individual Gaussians relative to the total area which was normalized to 1) as local parameters. The fitting model further included the modulation depth (local) to account for the inversion efficiency to the DEER signal (quantitative labeling of the protein was ensured by CW-EPR before PL incorporation). Protein concentration in the samples was <5 μM, thus intermolecular background contributions to the DEER signals were negligible. In order to avoid overfitting of the data we compared fits including a variable number of Gaussians using Akaike weights. All fits were run using the ‘MultiStart = 50’ option to avoid local minima. Reported errors reflect 50% and 95% confidence intervals for dipolar signals and distance distributions, which were calculated using the bootstrapping method (‘bootan’ function implemented in DEERlab) with 1000 iterations.

### Gating charge calculations

The electrostatic free energy of a protein in a membrane can be split into a membrane voltage-dependent part, and a voltage-independent reaction field free energy. Following the work of Roux^34^, the partial charges on the protein are the only explicit charges in the system. Thus, the membrane voltage-dependent part of the free energy can be calculated by zeroing all protein partial charges, calculating the potential everywhere with far-field boundary conditions that mimic the applied voltage, turning the partial charges back on, and summing the explicit charges multiplied by the potential over all charges in the protein. Specifically, we computed the voltage dependence of the transition from the inward to outward-facing conformations using the program APBSmem v 2.1.0^35^ to embed the proteins in a membrane-like environment and apply membrane voltage boundary conditions. One modification not described elsewhere is that in APBSmem v 2.1.0, protein cavities on the cytoplasmic (negative z) side of the membrane are filled with an effective charge density used to apply the membrane potential as described mathematically in Refs. ^33^ and ^35^. The electrostatics calculations were performed with APBSmem^36^ along with the PARSE parameter set for the protein charges. Briefly, for the two conformations, we computed the difference in the total interaction energy of all charges in the system with the membrane voltage between both states over a series of membrane potentials^37^, and then we extracted the equivalent total “gating charge” movement, as well as per residue contributions, from the slope of the line fitted to the resulting energy curve. For the inward-facing state, we used the inward-facing apo structure^4^, and we modeled the outward-facing conformation based on the SiaT structure from *P. mirabilis*, which shares ~24% sequence identity and ~46% sequence similarity with vSGLT. This latter model was created as described in Ref. ^7^. All calculation parameters and a rationale for their choices are provided in Supplementary Tables 4, 5 and discussed in greater detail in Refs. ^35,36^.

### Kinetic model of vSGLT transport

Kinetic equations were adapted from our 5-state stochastic release model presented in Refs. ^5^ and ^7^. The previous vSGLT transport parameters were largely adapted from experiments based on the human hSGLT1 homolog having little data to calibrate rates of the bacterial protein. We adjusted specific values to better fit the magnitude and shape of the single-transporter uptake rates provided in Fig. 2b and the observed ratios of inward-to-outward facing populations at different voltages in the DEER studies. Notably, four primary modifications to the rates were made. First, to match our DEER data, the rates of the inward-facing apo to outward-facing apo transition (k_51_, k_15_) were slowed and extracellular Na^+^ unbinding transition (k_21_) increased, effectively biasing the system towards the inward-facing state in the absence of voltage (Supplementary Table 6). Accordingly, the substrate rebinding rate (k_43_) and the reverse transport rate (k_32_) were slowed. Second, our gating charge calculations were based on the full inward-to-outward transition and thus provide estimates of the total charge transfer, but not on the fractions of charge transferred between states 2 to 3 and state 3 to 4. Without an occluded state structure (state 3), it is hard to estimate these values, but we assumed that the majority of the transfer occurs during inner gate opening (Supplementary Table 6). Third, we included a direct effect of Na^+^ binding (state 2) on charge transfer, as we hypothesize that a strong electric field from the Na^+^ ion as it is binding will bias the position of the gating charge residues in the membrane electric field even in the absence of a significant backbone conformational change. Fourth, we reduced the forward rate of Na^+^ binding from the extracellular space (k_12_) in order to match the magnitude of the experimentally measured uptake rates.

The full set of ordinary differential equations (ODEs) for vSGLT in the *normal* orientation is:

3 $$\frac{d{C}_{1}}{{dt}}=-\left({k}_{15}+{k}_{12}{[{Na}]}_{o}\right){C}_{1}+{k}_{21}{C}_{2}+{k}_{51}{C}_{5}$$

4 $$\frac{d{C}_{2}}{{dt}}={k}_{12}{{[{Na}]}_{o}C}_{1}-\left({k}_{21}+{k}_{23}{[G]}_{o}\right){C}_{2}+{k}_{32}{C}_{3}$$

5 $$\frac{d{C}_{3}}{{dt}}={k}_{23}{{[G]}_{o}C}_{2}-\left({k}_{32}+{k}_{34}\right){C}_{3}+{k}_{43}{C}_{4}$$

6 $$\frac{d{C}_{4}}{{dt}}={k}_{34}{C}_{3}-\left({k}_{43}+{k}_{45}\right){C}_{4}+{k}_{54}{[G]}_{i}{[{Na}]}_{i}{C}_{5}$$

7 $$\frac{d{C}_{5}}{{dt}}={k}_{15}{C}_{1}+{k}_{46}{C}_{4}-\left({k}_{54}{[G]}_{i}{[{Na}]}_{i}+{k}_{51}\right){C}_{5}$$

where the i subscript indicates intracellular values and the o subscript represents extracellular values, [Na] and [G] are the sodium and glucose Molar concentrations, respectively, and C_1-5_ are the time-dependent populations of the transporter in the different conformational states shown in Fig. 6a. The rate constants depend on membrane voltage according to:

8 $$\,{k}_{{ij}}={k}_{{ij}}^{0}\exp (-({\eta }_{{ij}}+{\epsilon }_{{ij}}){FV}/{RT})$$

where the zero superscript indicates the base rate constants shown in Supplementary Table 6 and (ε_ij_ – ε_ji_) is the equivalent Na^+^ charge movement from state i to j, while (η_ij_ – η_ji_) is the corresponding protein gating charge movement between states i and j. We assume that the voltage dependence of the charge transfer influences the forward and reverse rates equally. The corresponding set of differential equations for the *flipped* conformation is:

9 $$\frac{d{C^{\prime} }_{1}}{{dt}}=-\left({k^{\prime} }_{15}+{k^{\prime} }_{12}{[{Na}]}_{i}\right){C^{\prime} }_{1}+{k^{\prime} }_{21}{C^{\prime} }_{2}+{k^{\prime} }_{51}{C^{\prime} }_{5}$$

10 $$\frac{d{C^{\prime} }_{2}}{{dt}}={k^{\prime} }_{12}{[{Na}]}_{i}{C^{\prime} }_{1}-\left({k^{\prime} }_{21}+{k^{\prime} }_{23}{[G]}_{i}\right){C^{\prime} }_{2}+{k^{\prime} }_{32}{C^{\prime} }_{3}$$

11 $$\frac{d{C^{\prime} }_{3}}{{dt}}={k^{\prime} }_{23}{[G]}_{i}{C^{\prime} }_{2}-\left({k^{\prime} }_{32}+{k^{\prime} }_{34}\right){C^{\prime} }_{3}+{k^{\prime} }_{43}{C^{\prime} }_{4}$$

12 $$\frac{d{C^{\prime} }_{4}}{{dt}}={k^{\prime} }_{34}{C^{\prime} }_{3}-\left({k^{\prime} }_{43}+{k^{\prime} }_{45}\right){C^{\prime} }_{4}+{k^{\prime} }_{54}{[G]}_{o}{[{Na}]}_{o}{C^{\prime} }_{5}$$

13 $$\frac{d{C^{\prime} }_{5}}{{dt}}={k^{\prime} }_{15}{C^{\prime} }_{1}+{k^{\prime} }_{46}{C^{\prime} }_{4}-\left({k^{\prime} }_{54}{[G]}_{o}{[{Na}]}_{o}+{k^{\prime} }_{51}\right){C^{\prime} }_{5}$$

where the equations have an identical form to those for the normal orientation, but with the rate constants primed (*k* → *k*′) and the states replaced by primed values (C → C′). Additionally, the intracellular galactose and Na^+^ concentrations have been switched with their corresponding extracellular concentrations to indicate that they are binding/unbinding from different states in the flipped orientations. Finally, the primed rate constants have the same base rates (*k*^0^) but opposite dependence on voltage, since the flipped transporters experience the opposite membrane voltage:

14 $${k^{\prime} }_{{ij}}={k}_{{ij}}^{0}\exp (+({\eta }_{{ij}}+{\epsilon }_{{ij}}){FV}/{RT})$$

The analysis presented here only relies upon the steady state probability of the transporter in each state C_1_-C_5_ and C′_1_-C′_5_. We determined these values by numerically solving the ODEs until steady state was reached using the Rosenbrock stiff solver implemented in Berkeley Madonna^38^.

### Simulation of vSGLT

The outward-facing vSGLT conformation was embedded in a pure 1-Palmitoyl-2-Oleoyl-sn-Glycero-3-Phosphoethanolamine (POPE) lipid bilayer (344 total lipid molecules, 172 in each leaflet) and solvated with neutralizing 0.15 M NaCl in a box size of 11x11x11 nm^3^ using CHARMM-GUI^39–41^. To generate sodium-bound structures, a sodium ion was manually placed in the binding site, using the sodium-bound inward-facing structure (PDB ID: 3DH4) after backbone alignment of sodium site residues (62, 64, 361, 364 and 365) as a guide. The sidechains of 364 and 365 residues were manually adjusted to better match their orientation in the sodium-bound structure.

Simulations were performed with Gromacs 2020.6^42^ and the CHARMM-36m^43^ forcefield, with TIP3P water (25958 total water molecules; total atom count with all components 129462). Energy minimization and a multi-step equilibration, gradually reducing restraints on heavy protein and lipid atoms (and the bound sodium), was performed over 5 ns. A simulation timestep of 2 fs was used. Temperature and pressure were maintained at 303.15 K and 1 atm using v-rescale and a Parrinello-Rahman semi-isotropic barostat^44^, respectively. Non-bonded interactions were cut off at 1.2 nm, using force-switching from 1 nm. Long range electrostatic interactions were treated using the Particle-Mesh Ewald method^45^. Hydrogens were constrained using the LINCS algorithm^46^.

To explore conformational changes with and without sodium bound, three unbiased 100 ns simulations, with different initial velocities, were performed for each *apo* and sodium-bound structures. Analysis was performed using MDAnalysis 2.0^47,48^. Tilt of TM1a (resid 53-64) relative to the initial model was measured using helanal^49^, and distribution of E68 (in x, y and z) was measured as the distance of the final sidechain carbon (CD) from its position in the initial model (following alignment on carbon alpha atoms). Sodium distance was measured as the minimum distance of any sodium ion to the binding site (defined as the backbone center of mass of residues 62, 64, 361, 364, 365). For all three sodium-bound simulations, sodium was observed to unbind after ~15-50 ns; data was split into “sodium bound” and “sodium unbound” using sodium distance cutoffs of <2.5 Å and > 13 Å, respectively. Stability of the properties being measured over each simulation/configuration was checked by comparing block averages from the first and last 20 ns (unbound) or 5 ns (bound); differences were 1.0 ± 0.8 Å, 0.6 ± 0.4 Å, 0.6 ± 0.3 Å, and 1.8 ± 2.0^o^ for ∆x, ∆y, ∆z and tilt, respectively.

### Reporting summary

Further information on research design is available in the Nature Portfolio Reporting Summary linked to this article.

### Supplementary information

Supplementary Information

Reporting Summary

### Source data

Source Data

## Data Availability

The galactose uptake data and the DEER data generated in this study have been deposited in the Zenodo database under accession code 10.5281/zenodo.8431614. Source data are provided as a Source Data file. Source data are provided with this paper.

## Code availability

Input and coordinate files (initial and final) for molecular dynamics production runs have been deposited in the Zenodo database under accession code 10.5281/zenodo.10000256, along with code for the 5 state model and gating charge calculations. All other codes developed for this paper will be made available upon request.
