# Supplementary material for: Membrane potential accelerates sugar uptake by stabilizing the outward facing conformation of the Na/glucose symporter vSGLT
Source: Nat Commun. 2023 Nov 18;14:7511. doi: 10.1038/s41467-023-43119-z (PMC10657379; doi:10.1038/s41467-023-43119-z)
Supplement: Supplementary file 1 — Supplementary Information [file 41467_2023_43119_MOESM1_ESM.pdf]

Supplementary material for:

Negative membrane potential accelerates sugar uptake by stabilizing the outward-facing conformation of the Na/glucose symporter vSGLT

Farha Khan[1,6,7], Matthias Elgeti[2,4,6,10], Samuel Grandfield[1,8], Aviv Paz[1,9], Fiona B. Naughton[5], Frank V. Marcoline[5], Thorsten Althoff[1], Natalia Ermolova[1], Ernest M. Wright[1], Wayne L. Hubbell[2,3], Michael Grabe[5,10], Jeff Abramson[1,10]

[1]Department of Physiology, David Geffen School of Medicine; [2]Jules Stein Eye Institute [3]Department of Chemistry and Biochemistry; University of California, Los Angeles, Los Angeles, CA 90095, USA. [4]Institute for Drug Discovery, Leipzig University Medical School, Leipzig, Germany. [5]Department of Pharmaceutical Chemistry, Cardiovascular Research Institute, University of California, San Francisco, San Francisco, CA 94158, USA.

[6] equal contributions

[7] Present Address: Department of Structural Biology, Van Andel Institute, Grand Rapids, MI, 49503, USA

[8] Present Address: Renaissance School of Medicine at Stony Brook University, Stony Brook, New York, 11794, USA

[9] Present address: Hauptman-Woodward Medical Research Institute, 700 Ellicott Street, Buffalo, New York 14203, USA

[10] To whom correspondence should be addressed: Matthias Elgeti (melgeti@ucla.edu), Michael Grabe (michael.grabe@ucsf.edu), and Jeff Abramson (jabramson@mednet.ucla.edu).

Includes:

Supplementary figures 1 to 8

Supplementary table 1 to 6

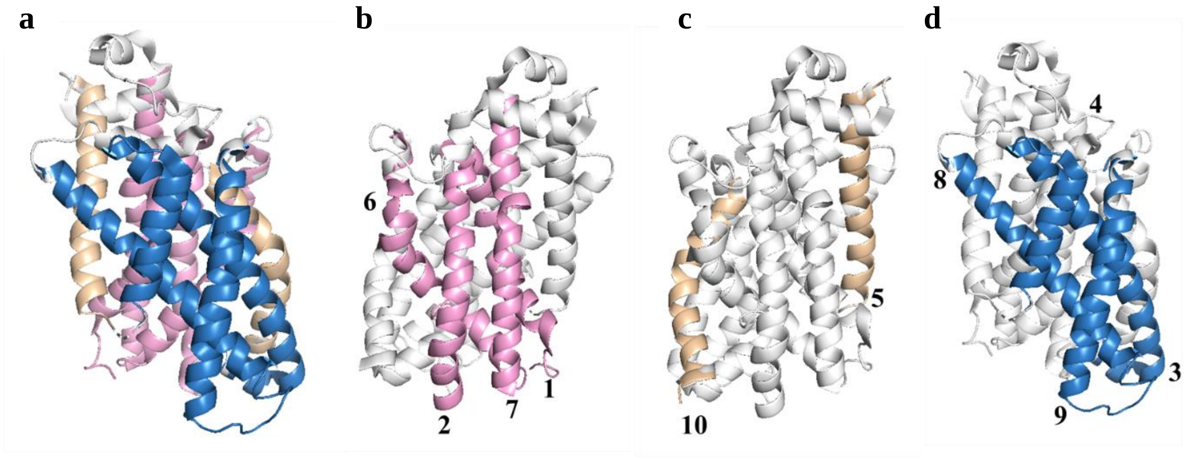

Supplementary Fig. 1: The arrangement of vSGLT's domains. (a) The 5TMIR core can be divided into a bundle domain (pink) and a scaffold domain (blue and brown). **(b)** The bundle domain (pink). The Scaffold domain can be further subdivided into the **(c)** gating helices (brown) and **(d)** hash motif (blue). The TM helix numbers are labeled next to the helices.

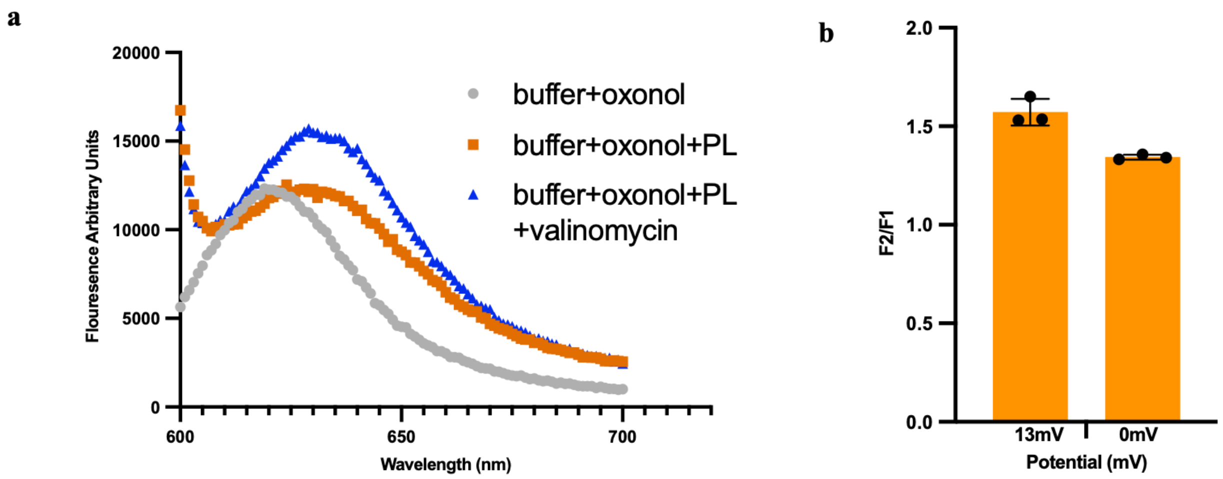

Supplementary Fig. 2: The *E. coli* lipidic PL system shows voltage-dependent fluorescence changes with the use of Oxonol VI. To confirm the generation of voltages in the PL system, Oxonol VI, a voltage-sensitive dye, was employed due to its ability to exhibit fluorescence shifts in response to inside-positive transmembrane voltage. Oxonol VI was employed in the presence and absence of valinomycin. **(a)** In buffer, Oxonol VI has an emission peak at 620 nm (blue trace), which undergoes a red-shift and a slight amplitude increase upon the addition of PLs (orange trace). The introduction of valinomycin-induced TMP results in a more pronounced amplitude rise with a concurrent minor red-shift (gray trace). The data depicted represents a typical plot for the +13mV condition. **(b)** The peak amplitude ratios at 630 nm for the PL and valinomycin condition divided by the amplitude of the PL condition are plotted at positive (+13mV) and zero TMP. Data represented here is from three sets of independent triplicates for each voltage, error bars represent standard deviation.

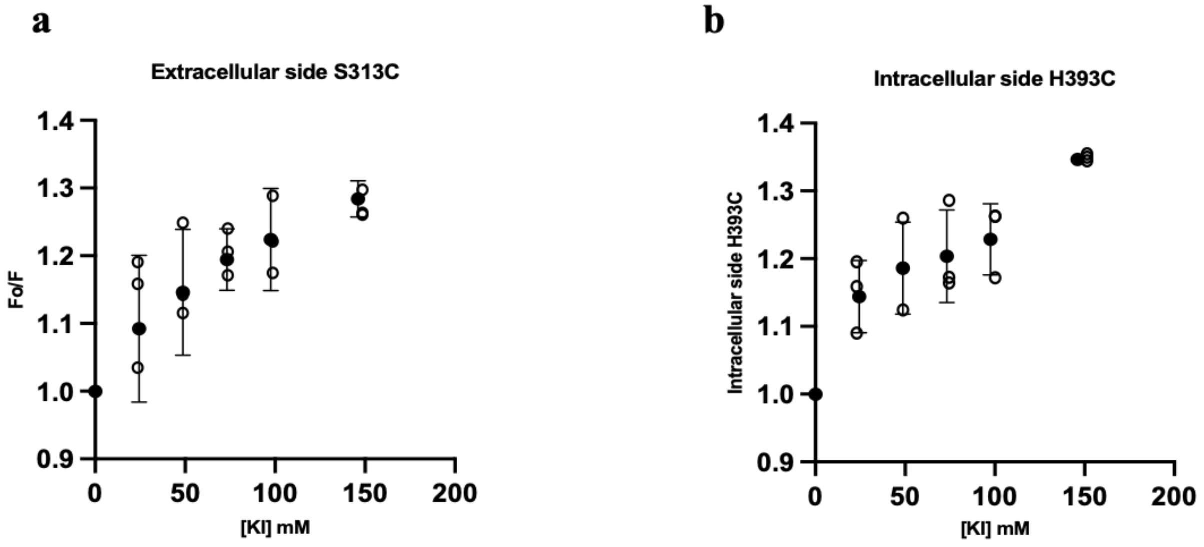

Supplementary Fig. 3: Fluorescence Quenching of Pyrene-Labeled vSGLT in reconstituted PLs. To ascertain the orientation of vSGLT within PLs, pyrene-maleimide was utilized for labeling vSGLT mutants S313C and H393C, representing residues facing the intracellular and extracellular sides of the membrane, respectively. These labeled proteins were reconstituted in PLs, and potassium iodide was employed to quench the fluorescence signal of **(a)** 313-pyrene and **(b)** 393-pyrene. Notably, fluorescence quenching was exclusively observed in the labeled mutants exposed on the outer surface of PLs. Both sites demonstrated comparable Stern-Volmer quenching constants and linear fits of the quenching data, suggesting that vSGLT incorporates into PLs in both directions with equal propensity. Data represented here is from three sets of independent triplicates, error bars represent standard deviation.

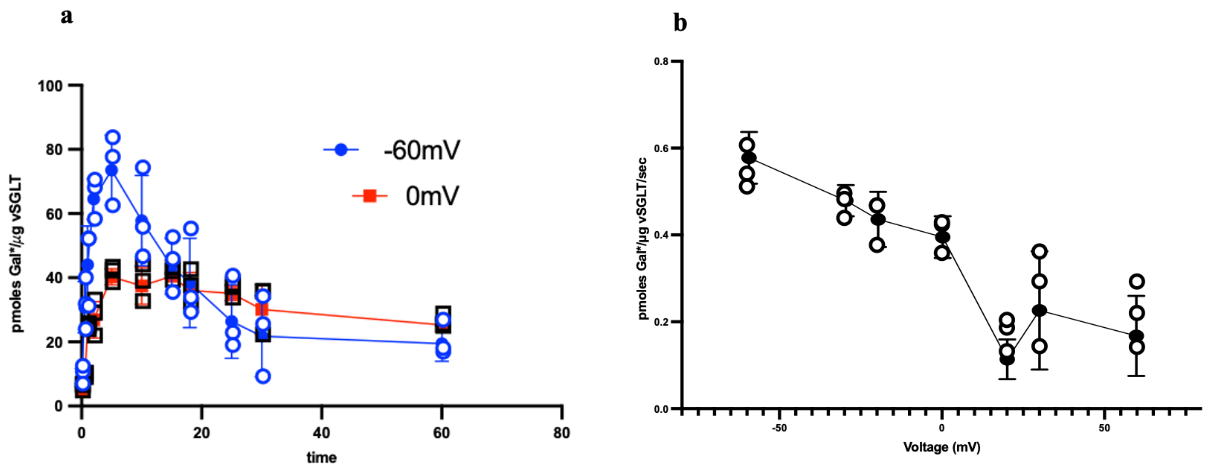

Supplementary Fig. 4: Influence of Positive TMP on Sugar Uptake Rates.

(a) Comprehensive time course of sugar uptake at 0 mV and -60 mV. **(b)** To investigate the impact of sugar uptake under positive membrane potentials, an alternative buffering system incorporating choline was employed (see methods). When utilizing choline chloride, the efficiency of the transport assay decreased (compared to uptake values obtained from the original buffering system). Nevertheless, in line with the original assay (Figure 2), an enhancement in sugar transport becomes apparent with increasingly negative TMPs. The introduction of a positive membrane potential leads to a saturation point in uptake, displaying non-linear behavior. Data represented here is from three sets of independent triplicates for each voltage, error bars represent standard deviation.

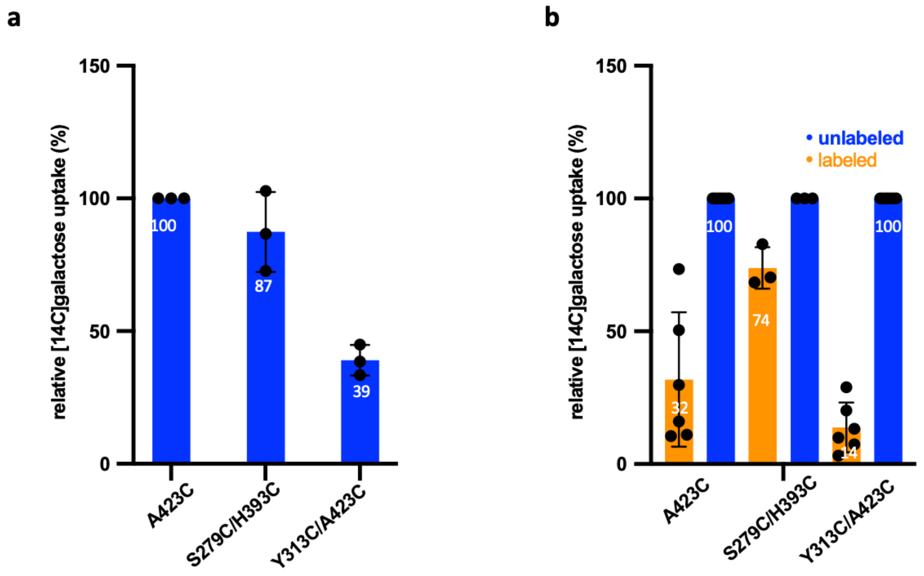

Supplementary Fig. 5: Assessment of the transport activity for the mutant vSGLT constructs. **(a)** The activity of vSGLT constructs employed for DEER studies was evaluated by sugar uptake assay and compared with the wild type vSGLT (A423C). **(b)** The activity of the labeled mutants normalized to their respective unlabeled proteins. Data represented here is from three or more sets of independent triplicates for each construct, error bars represent standard deviation.

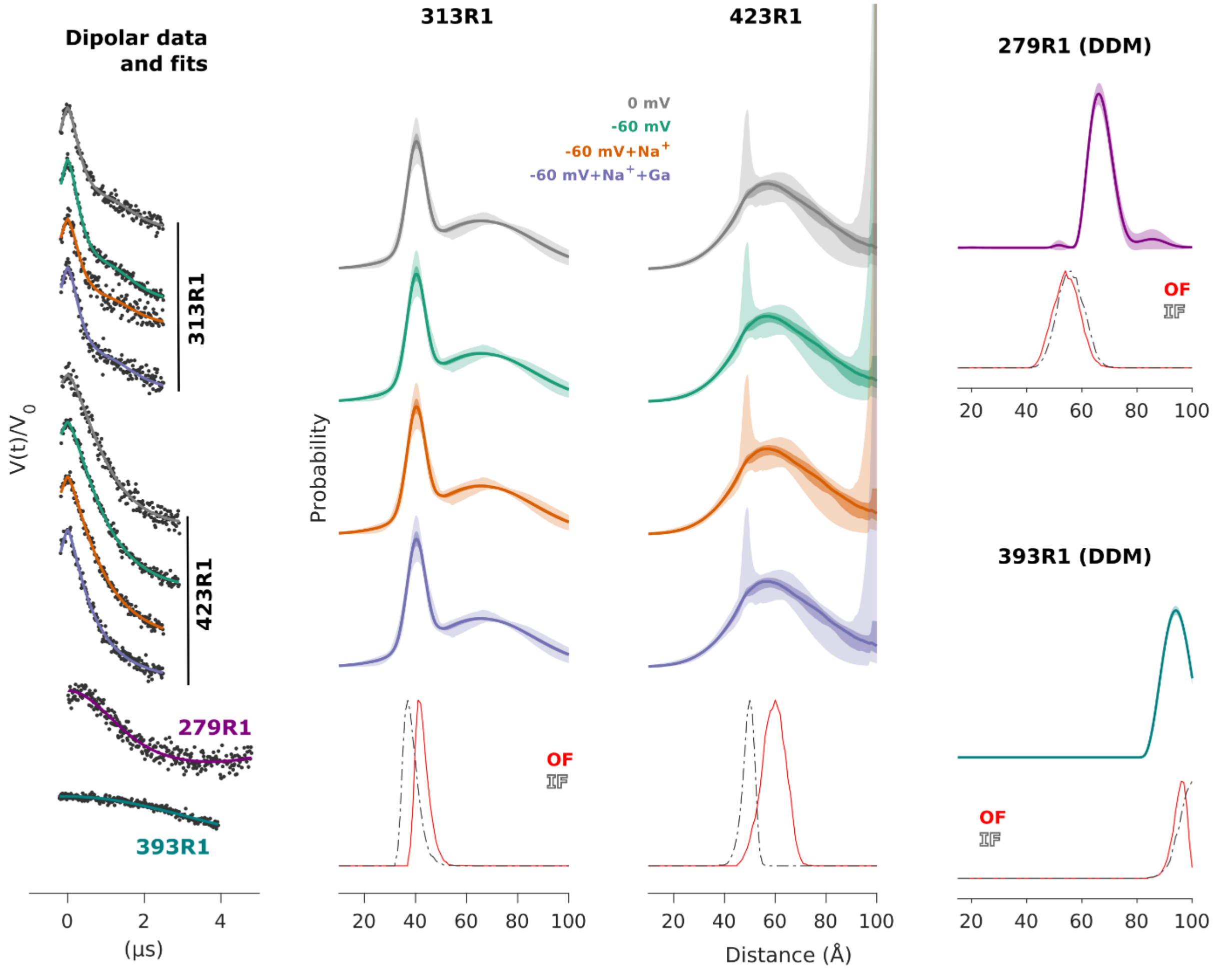

Supplementary Fig. 6: vSGLT dimer distances. For both DEER mutants, 313R1/423R1 and 279R1/393R1, the increased modulation depth (~60%) and the presence of long distances indicates the existence of vSGLT dimers, which has been previously reported for solubilized vSGLT [7]. To verify the dimer observed in crystal structures (PDB ID: 2XQ2) or the SiaT derived model of the outward facing conformation, we performed DEER on singly labeled vSGLT and compared these with MDDS derived distance distributions. Note that because of the low protein concentration (< 5 μM) DEER distances for 279R1 and 393R1 were too long to be captured in our PL system. However, we were able to capture these distances in detergent micelles in the presence of 20% deuterated glycerol. 50% and 95% confidence bands are shown with increasing transparency.

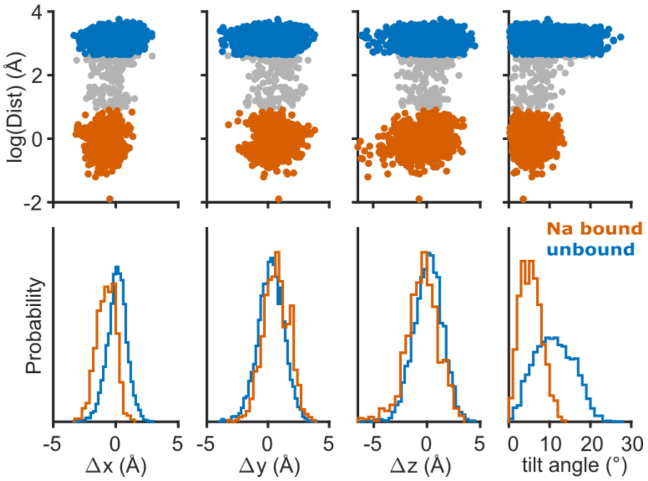

Supplementary Fig. 7: Outer gate movement in response to Na^+ binding: The change in the distance distribution of E68 and tilt of the helix TM1a with Na^+ bound or unbound, as observed by MD simulations. Shown top against the minimum Na^+-binding site distance, and bottom as histogram. Sodium distance cut-offs of < 2.5 Å and > 13 Å were used to define "bound" and "unbound". Three simulations were performed starting in each the *apo* and sodium-bound conditions; histograms shown collect all simulation data. Average overlap (histogram intersection) between data from individual simulations for Δx is 0.72 ± 0.13 when comparing bound vs. bound (three repeats) or unbound vs. unbound (six repeats – three starting unbound, three repeats after sodium unbinds from an initially bound state) and 0.47 ± 0.2 when comparing bound vs. unbound. For Δy, the same figures are 0.72 ± 0.13 and 0.69 ± 0.16, respectively; for Δz: 0.77 ± 0.07 and 0.72 ± 0.1; and for tilt angle: 0.65 ± 0.14 and 0.44 ± 0.12.

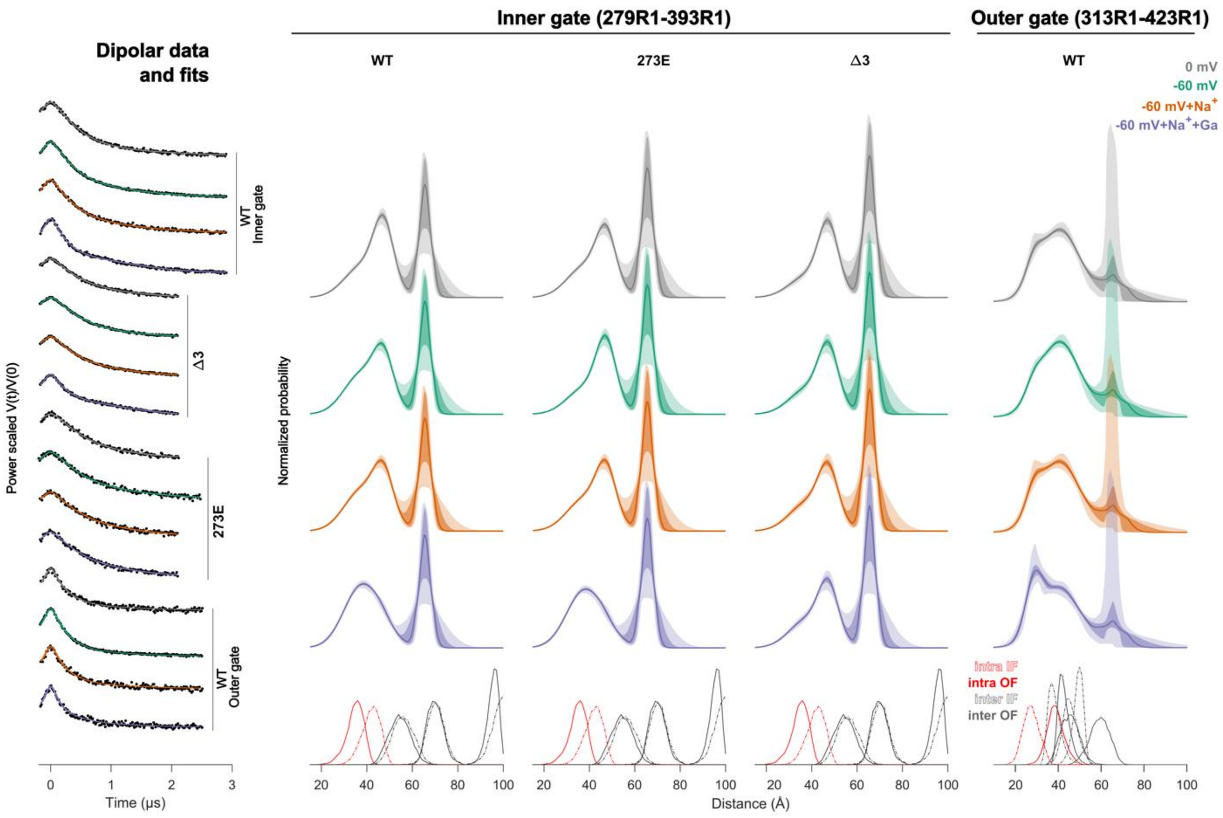

Supplementary Fig. 8: DEER analysis of 279R1/393R1, 279R1/393R1-273E, 279R1/393R1-Δ3 and 313R1/423R1 constructs. Note, that all samples with 279R1/393R1 background were analyzed globally. For each pair the MDDS derived intra- (red) and intermolecular (black) distances are shown for comparison. 50% and 95% confidence bands are shown with increasing transparency.

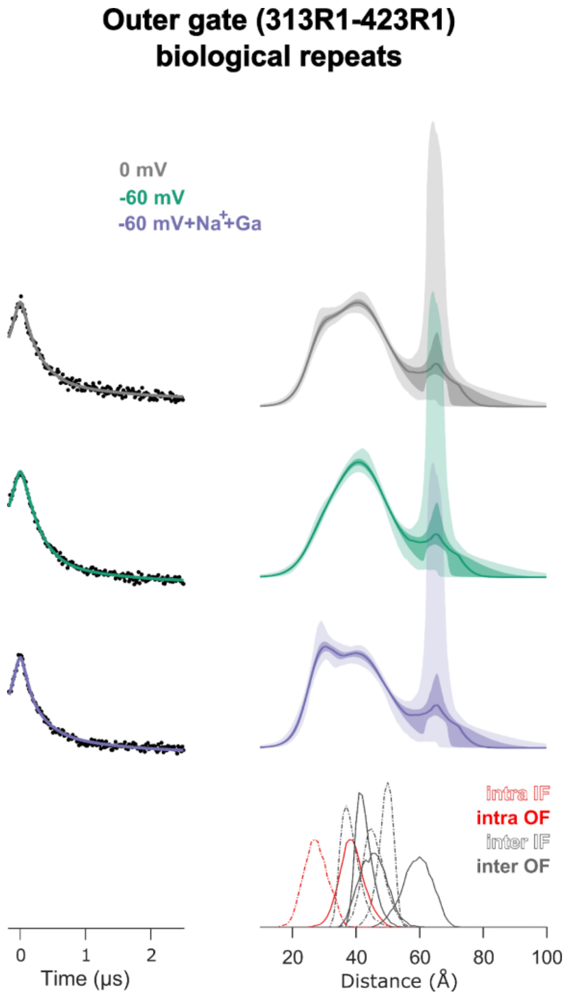

Supplementary Fig. 9: Biological repeats of 313R1/423R1. Opening and closure of the outer gate under TMP of -60 mV and in the presence of substrate, respectively. Modeled distance distributions for comparison. 50% and 95% confidence bands are shown with increasing transparency.

Voltage (mV)	K^+ inside (mM)	K^+ outside (mM)	Na^+ outside (mM)
0	150	75	73
-5	150	119	73
-11	150	95	73
-17	150	75	73
-21	150	65	72
-27	150	53	72
-31	150	43	72
-38	150	33	73
-48	150	23	73
-60	150	75	73

Supplementary Table 1: Buffer conditions for inducing negative TMPs.

Voltage (mV)	K^+ inside (mM)	K^+ outside (mM)	Na^+ outside (mM)
-60	1	0.1	73
-30	1	0.3	73
-20	1	0.4	73
0	1	0.1	74
20	1	2.1	73
30	1	3.2	73
60	1	11.0	73

Supplementary Table 2: Buffer conditions for inducing positive TMPs.

Sample	[KI] (M)	[KCl] (M)	[$Na_2S_2O_3$] (M)
A	0.9	0.0	10^{-5}
B	0.6	0.3	10^{-5}
C	0.45	0.45	10^{-5}
D	0.3	0.6	10^{-5}
E	0.15	0.75	10^{-5}

Supplementary Table 3: Buffer conditions for the quenching experiments

Residue	Gating charge
E68	-0.61
E88	-0.44
D189	-0.34
R273	0.32
K294	0.28
K471	0.25
D336	-0.17
D481	-0.13
D12	-0.10
D380	-0.10

Supplementary Table 4: List of top 10 residues contributing to the gating charge.

Description	Value
charge model	PARSE
counter ions	±1 e, 0.1 M, 2.0 Å
temperature	298.15 K
grid dimensions	225×225×225
coarse grid size	300×300×300 $Å^3$
fine grid size	75×75×75 $Å^3$
protein dielectric	2
membrane dielectric	2
headgroup dielectric	80
solvent dielectric	80
hydrophobic thickness	30 Å
headgroup thickness	8 Å
grid center	origin
solution method	lpbe
boundary condition	memv
charge model	spl2
surface model	mol
solvent probe radius	1.4 Å
surface sphere density	10 $Å^{-2}$

Supplementary Table 5: Parameters for electrostatic calculations.

Parameter	vSGLT	Gating charge transfer fraction	Na^+ ion transfer fraction
k_{12} [$M^{-1}\cdot s^{-1}$]	2,000	$\eta_{12} = 0.05 \cdot Q_{gc}$	$\varepsilon_{12} = 0.23 \cdot 1$
k_{21} [s^{-1}]	1,250	$\eta_{21} = -0.05 \cdot Q_{gc}$	$\varepsilon_{21} = -0.23 \cdot 1$
k_{23} [$M^{-1}\cdot s^{-1}$]	45,000	$\eta_{23} = 0.05 \cdot Q_{gc}$	$\varepsilon_{23} = 0.0575 \cdot 1$
k_{32} [s^{-1}]	5	$\eta_{32} = -0.05 \cdot Q_{gc}$	$\varepsilon_{32} = -0.0575 \cdot 1$
k_{34} [s^{-1}]	50	$\eta_{34} = 0.35 \cdot Q_{gc}$	$\varepsilon_{34} = 0.0575 \cdot 1$
k_{43} [s^{-1}]	20	$\eta_{43} = -0.35 \cdot Q_{gc}$	$\varepsilon_{43} = -0.0575 \cdot 1$
k_{45} [s^{-1}]	10	$\eta_{45} = 0.05 \cdot Q_{gc}$	$\varepsilon_{45} = 0.155 \cdot 1$
k_{54} [$M^{-2}\cdot s^{-1}$]	1.08E5	$\eta_{54} = -0.05 \cdot Q_{gc}$	$\varepsilon_{54} = -0.155 \cdot 1$
k_{51} [s^{-1}]	6	$\eta_{51} = -0.50 \cdot Q_{gc}$	$\varepsilon_{51} = 0$
k_{15} [s^{-1}]	20	$\eta_{15} = 0.50 \cdot Q_{gc}$	$\varepsilon_{15} = 0$

Supplementary Table 6: Kinetic parameters for the transport model. Note that in our convention, the gating charge transfer for the protein or ion between states i and j is ($\eta_{ij} - \eta_{ji}$) or ($\varepsilon_{ij} - \varepsilon_{ji}$), respectively, and the impact of the charge on the forward and reverse rates are split evenly among the forward/reverse rates. For most calculations, $Q_{gc} = -0.7$, but it is also set to 0 and +0.7 where indicated.
